# Hygienic Importance of Light

**Published:** 1863-11

**Authors:** 


Hygienic Importance of Light.—Dr. Moore, the metaphysician, thus speaks of the effect of light on body and mind:—"A tadpold confined in darkness would never become a frog; and an infant being deprived of heaven's free light will only grow into a shapeless idiot, instead of a beautiful and responsible being. Hence, in the deep, dark gorges and ravines of Swiss Valais, where the direct sunshine never reaches, the hideous prevalence of idiocy startles the traveler. It is a strange melancholy idiocy, many citizens are incapable of articulate speech, some are deaf, some are blind, some labor under all these privations, and all are misshapen in almost every part of the body. I believe there is in all places a marked difference in the healthiness of houses according to their aspect with regard to the sun, and those are decidedly the healthiest, other things being equal, in which all the rooms are, during some parts of the day fully exposed to the light.

Epidemics attack inhabitants on the shady side of the street and exempt those on the other side, and even in epidemics, such as ague, the morbid influence is often thus partial in its labors.

